# Linking meniscal pathology to Baker’s cyst formation: the role of tear type, location and chondral damage

**DOI:** 10.1186/s12891-026-09896-6

**Published:** 2026-05-01

**Authors:** Oguzhan Ak, Muhammet Baybars Ataoglu, Ethem Burak Oklaz, Ramazan Duzgun, Muhammed Sakir Calta, Elif Banu Guler Oklaz, Ulunay Kanatli

**Affiliations:** 1 Department of Orthopaedics and Traumatology, Sungurlu State Hospital, Corum, Turkey; 2 Department of Orthopaedics and Traumatology, Gazi University Faculty of Medicine, Ankara, Turkey; 3 Department of Orthopaedics and Traumatology, Dogubayazit Dr. Yasar Eryilmaz State Hospital, Agri, Turkey; 4 Department of Orthopaedics and Traumatology, Nevsehir State Hospital, Nevsehir, Turkey; 5 Department of Orthopaedics and Traumatology, Cubuk Halil Sivgin State Hospital, Ankara, Turkey; 6 Department of Radiology, Dogubayazit Dr. Yasar Eryilmaz State Hospital, Agri, Turkey

**Keywords:** Baker’s cyst, popliteal cyst, Cooper classification, tear pattern, meniscus

## Abstract

**Background:**

Although Baker’s cysts are commonly associated with meniscal tears, the role of specific tear morphologies and anatomic locations in cyst presence remains unclear. This study aimed to evaluate the associations between meniscal tear patterns, tear locations, and Baker’s cysts and to explore whether these tear characteristics were also associated with concomitant chondral lesions.

**Methods:**

In this retrospective cohort study, patients who underwent knee arthroscopy at a single institution were evaluated and categorized according to the presence or absence of a Baker’s cyst on MRI. Arthroscopic video recordings were reviewed to identify meniscal tear morphology and tear location based on the Cooper classification, as well as the presence of chondral lesions. Separate multivariate logistic regression models were constructed for tear morphology and tear location to account for collinearity among meniscal variables.

**Results:**

Of 353 patients (mean age 35.5 ± 13.4 years), 77 (21.8%) had a Baker’s cyst. In the multivariate tear morphology model, horizontal, radial, and complex medial meniscal tears were significantly associated with Baker’s cysts (OR: 7.321, 95% CI: 2.921–18.349, p < 0.001; OR: 3.380, 95% CI: 1.136–10.056, p = 0.039; and OR: 4.000, 95% CI: 1.907–8.394, p < 0.001, respectively). In the multivariate location model, tears involving Cooper zones A2, A3, and B3 were also significantly associated with Baker’s cysts (OR: 2.956, 95% CI: 1.133–7.713, p = 0.027; OR: 3.978, 95% CI: 1.838–8.606, p < 0.001; and OR: 7.070, 95% CI: 2.878–17.371, p < 0.001, respectively). Chondral lesions remained independently associated with Baker’s cysts in both models. In univariate analyses, these tear types and locations were also associated with chondral lesions, although these associations were not maintained after multivariate adjustment.

**Conclusion:**

In this retrospective cohort, horizontal, radial, and complex medial meniscal tears, particularly those involving Cooper zones A2, A3, and B3, were associated with the presence of Baker’s cysts. These findings expand the current literature by providing a more detailed description of the relationship between meniscal tear morphology, tear location, and Baker’s cysts, and may serve as a basis for future studies investigating the underlying mechanisms.

## Background

Baker’s cysts, or popliteal cysts, are frequently encountered in orthopedic practice, and in adults, Baker’s cysts are often linked to underlying intra-articular conditions, most notably meniscal tears [30, 33]. Additional contributing factors include osteoarthritis, chondral lesions, anterior cruciate ligament (ACL) injuries, and systemic inflammatory diseases [2, 24, 25, 30, 34]. A substantial proportion of these cysts are asymptomatic and incidentally detected on imaging, with reported prevalence ranging from 4.7% to 37% in various populations. This variability highlights the clinical challenge of distinguishing incidental findings from cysts that reflect clinically meaningful intra-articular pathology [16, 17, 19]. 

Meniscal tears associated with Baker’s cysts are typically located in the medial meniscus [3, 33]. Despite the high prevalence of these tears in patients with Baker’s cysts, the precise mechanisms underlying this association remain poorly understood. Two commonly proposed mechanisms for cyst formation include increased intra-articular pressure leading to synovial fluid distension of the gastrocnemius–semimembranosus bursa, and altered joint biomechanics facilitating fluid passage through a valvular communication into the bursa [17, 19, 32]. 

To effectively prevent or treat symptomatic Baker’s cysts, a deeper understanding of the meniscal pathologies associated with their development is required. Identifying which meniscal tear types and anatomic locations predispose to Baker’s cyst formation may refine pathophysiological models and help guide treatment strategies. Therefore, this study aimed to clarify the impact of meniscal tear patterns and locations on Baker’s cyst formation through arthroscopic confirmation and systematic Cooper classification mapping, with the goal of providing a foundation for future approaches to Baker’s cyst management.

We hypothesized that certain tear morphologies and Cooper zones may be more frequently associated with Baker’s cysts. A secondary aim was to explore whether these tear types also tend to coexist with chondral lesions, suggesting a multifactorial mechanism in cyst development. The research questions this study aimed to answer were: (1) Do specific meniscal tear morphologies and locations linked to Baker’s cyst development? (2) Do these tear types and locations also predispose to chondral pathology, suggesting a multifactorial mechanism of cyst formation?

## Methods

### Study desing

This study was designed as a retrospective cohort study conducted at a single academic tertiary referral center. The initial dataset comprised 557 patients, of whom 353 met the eligibility criteria and were included in the final analysis. To determine whether the sample size was sufficient, a power analysis was conducted. An a priori power analysis was performed using the chi-square test family within the G*Power 3.1 software, selecting the Goodness-of-Fit statistical method. The alpha error probability (α) was 0.05, and the desired power was (1-β) 0.95. Therefore, the final sample size was considered adequate for the primary analyses, while acknowledging potential limitations for subgroup analyses.

Patients who were admitted to the institution with a diagnosis of knee pathologies and underwent arthroscopic surgery by two senior surgeons between January 2016 and January 2024 were retrospectively evaluated. Stringent exclusion criteria were applied to ensure data quality and homogeneity. Patients were excluded if they had systemic inflammatory diseases, pre-existing osteoarthritis, a history of prior knee arthroscopies, malignancies around the knee joint, knee infections, structural meniscal abnormalities such as a discoid meniscus, ruptured Baker’s cysts, or prior treatments specifically targeting Baker’s cysts. Pre-existing osteoarthritis was excluded using standard radiographic evaluation. Patients demonstrating radiographic findings such as joint space narrowing, osteophyte formation, subchondral sclerosis, or cyst formation were excluded from the study. Also, lower limb alignment parameters were not included in the analysis due to the lack of consistent radiographic data. These criteria were specifically applied to minimize the influence of degenerative and inflammatory confounders that could independently affect both meniscal pathology and Baker’s cyst formation. Additional exclusions included those with insufficient arthroscopic examination or poor-quality video recordings or MRI scans and patients under 18 years of age. De Maeseneer et al. reported that pediatric Baker’s cyst cases in patients aged 1 to 18 years were not associated with any meniscal tears; therefore, patients under the age of 18 were excluded from the study [10]. Following these exclusions, 353 patients remained and were included in the analysis.

### Arthroscopic evaluation

**Fig. 1:**
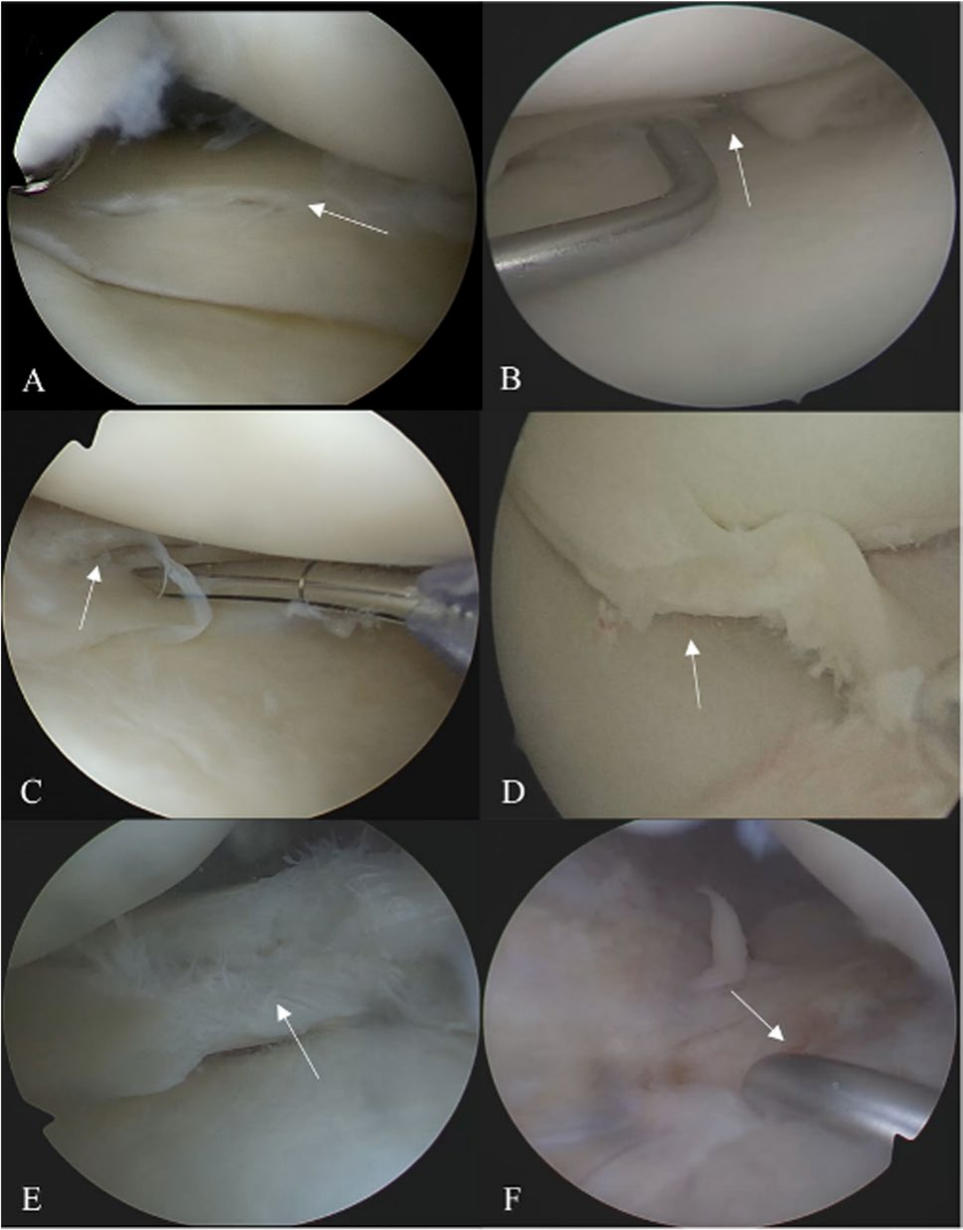
Arthroscopic images of the evaluated tear types are presented as follows: Horizontal meniscal tear (**a**), radial meniscal tear (**b**), vertical/longitudinal meniscal tear (**c**), bucket-handle meniscal tear (**d**), degenerative/complex meniscal tear (**e**), and ramp lesion (**f**). White arrow shows the tear location

**Fig. 2:**
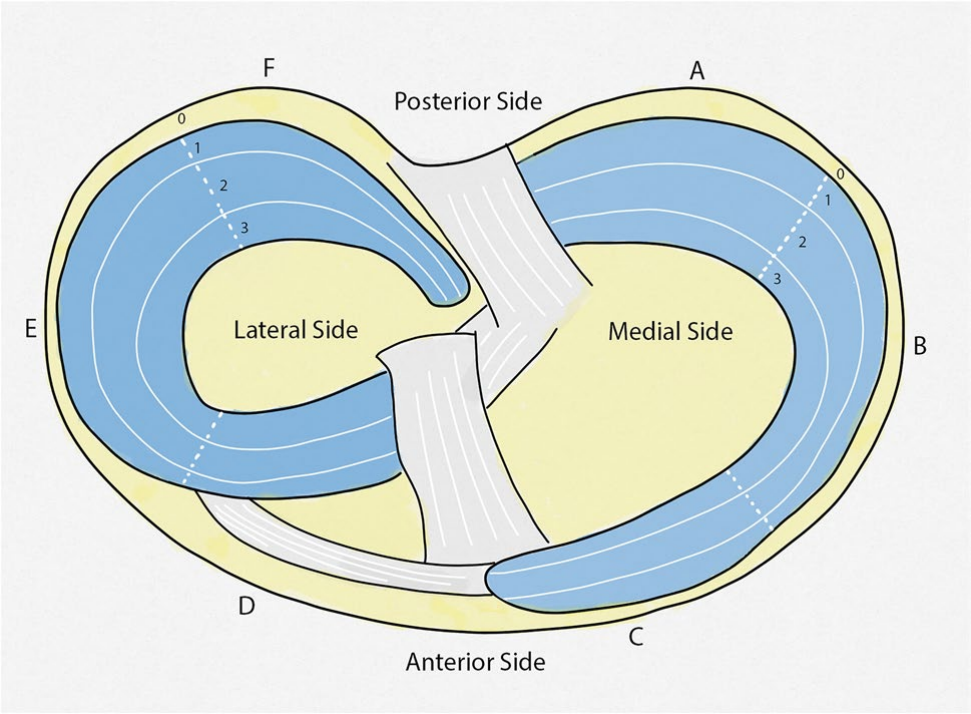
The meniscus is divided into radial and circumferential zones. Radial zones are labeled alphabetically (**A**–**F**). Circumferential zones are labeled numerically (0–3), extending from the meniscocapsular junction to the inner third of the meniscus

During arthroscopic examination, both menisci were systematically evaluated using a probe. Arthroscopy video recordings were reviewed, tear patterns and tear locations were meticulously recorded. Meniscal tear patterns were categorized into five types for both menisci: horizontal, radial, vertical, complex and bucket-handle tears. Complex tears were defined as tears exhibiting more than two distinct patterns or as highly degenerative tears where no specific pattern was evident. Specifically vertical tears at the capsular attachment of the posterior medial meniscus were specifically identified and documented as ramp lesions when they co-occurred with an anterior cruciate ligament (ACL) tear. Tear locations were documented using the Cooper classification system [9, 35]. According to this classification, the meniscus was divided into radial zones labeled A, B, C, D, E, and F, and circumferential zones numbered 0, 1, 2 and 3 extending from the meniscocapsular junction to the inner third of the meniscus. For tears involving multiple zones, the location was assigned to the zone with the largest tear width or the most severe structural disruption seen during arthroscopy. The presence of ACL tears and chondral lesions were also recorded. Arthroscopic images of tear patterns and ramp lesion are provided in Fig. 1 and an illustration of the Cooper classification is provided in Fig. 2.

Arthroscopic surgery video recordings were evaluated by two experienced orthopaedic surgeons. To investigate intraobserver reliability, the same observer reevaluated video records at intervals of more than 2 weeks from the initial evaluation. To evaluate interobserver reliability, another observer similarly evaluated all the video records.

### Radiological evaluation

**Fig. 3:**
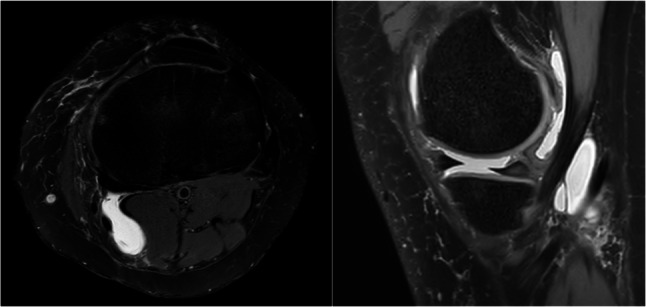
Sagittal and axial T2-weighted magnetic resonance images demonstrating a Baker’s cyst in the gastrocnemius–semimembranosus recess

To determine the presence or absence of Baker’s cysts, MRI images were evaluated, as MRI is considered the gold standard for this assessment. The imaging protocol included sagittal and axial T2-weighted sequences. Baker’s cysts were defined by their characteristic location in the gastrocnemius–semimembranosus recess on MRI. No minimum size threshold was applied, as no standard size criterion exists. Fluid collections within or adjacent to the meniscus without communication with this bursa were defined as parameniscal cysts and were not considered as Baker’s cysts in this study. [17] All MRI scans were independently evaluated by a musculoskeletal radiologist and an orthopaedic knee surgeon, both blinded to prior radiologic reports and arthroscopic findings. Representative MRI images demonstrating a Baker’s cyst are shown in Fig. 3.

### Statistical analysis

Statistical analyses were conducted using IBM SPSS Statistics Version 29.0.2.0 (SPSS, Chicago, IL, USA). Continuous variables were reported as means. Categorical variables were presented as frequencies and percentages. Given that the distributions of age were non-normal in Kolmogorov-Smirnov test, Mann-Whitney U test were employed for the appropriate statistical comparisons.

The association between meniscal tear patterns, tear locations, and the presence of Baker’s cysts was assessed using the chi-square test. Binary logistic regression analysis was performed to estimate odds ratios (ORs) with 95% confidence intervals (CIs) for factors associated with Baker’s cyst formation. To address potential multicollinearity among meniscal variables, separate multivariate models were constructed using alternative representations of meniscal pathology. Specifically, tear morphology and anatomical location (Cooper classification) were analyzed in independent models alongside age and chondral lesions. Exploratory analyses were additionally performed to evaluate the relationships between ACL injury and chondral lesions using regression models. A p-value < 0.05 was considered statistically significant.

Cohen’s kappa coefficient was used to assess intra-observer and inter-observer agreement for categorical variables. When evaluating consistency in tear types, the intra-observer kappa coefficient was found to be 0.886, while the inter-observer kappa coefficient was 0.889, indicating excellent agreement in both cases. Similarly, for tear regions, the intra-observer kappa coefficient was 0.875, and the inter-observer kappa coefficient was 0.914, demonstrating excellent consistency in both measurements. The results were interpreted using the Landis and Koch criteria, where values of 0.81-1.00 indicate excellent agreement.

## Results

**Fig. 4:**
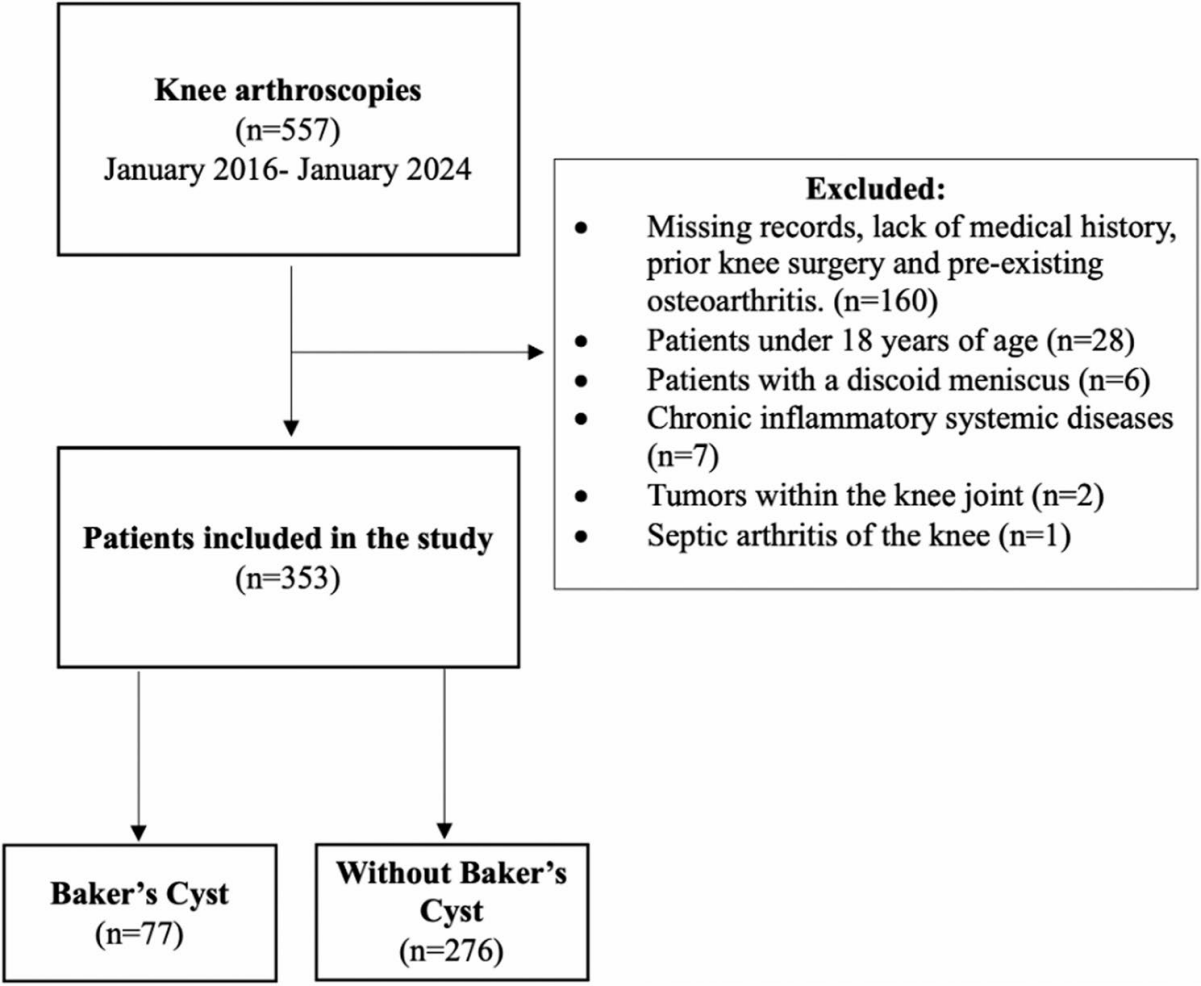
A flowchart of patient selection

Following application of the predefined inclusion and exclusion criteria, 353 patients were included in the final analysis; the patient selection process is summarized in Fig. 4.

The study population consisted of 130 females and 223 males. There was no statistically significant difference in sex distribution between patients with and without Baker’s cyst (*p* = 0.0661).

The mean age of the study population was 35.5 ± 13.43 years. A significant age difference was observed between the groups, with the Baker’s cyst group being significantly older than the without Baker’s cyst group (*p* = 0.002).

Meniscal tears significantly associated with the development of Baker’s cysts in the chi-square test, regardless of whether the tear was medial or lateral (*p* < 0.001).

ACL tears were not significantly associated with the development of Baker’s cysts (*p* = 0.231). Also specifically Ramp lesions were not significantly associated with the development of Baker’s cyst (*p* = 0.662).

Chondral lesions demonstrated a significant assosiation with Baker’s cysts in the chi-square test (*p* < 0.001). Demographics and general clinical data are demonstrated in Table 1.

**Table 1:** Demographics and general clinical data. Bold values indicate statistical significance at p < 0.05

Variable	Baker’s Cyst (*n* = 77)	Without Baker’s Cyst(*n* = 276)	*p*-value
Age (mean)	39.77 ± 13.98	34.29 ± 13.06	**0.002**
Sex (n,%)	Female: 30 (39%) Male 47(61%)	Female: 100 (36.2%) Male: 176 (63.8%)	0.0661
Meniscal Tear (n,%)	63 (81.8%)	133 (48.2%)	**< 0.001**
ACL Tear (n,%)	32 (41.6%)	126 (45.7%)	0.231
Chondral Lesion (n,%)	34 (44.2%)	35 (12,68%)	**< 0.001**
Ramp Lesion (n,%)	3 (3.9%)	8 (2.89%)	0.662

Meniscal tear patterns and tear locations were significantly associated with Baker’s cysts in the chi-square test. (*p* < 0.001, *p* < 0.001, respectively) Medial meniscal tear patterns were significantly associated with Baker’s cysts, whereas lateral meniscal tear patterns were not (*p* < 0.001, *p* = 0.643, respectively).

Meniscal tear patterns for both menisci and tear localizations for both menisci based on Cooper Classification are demonstrated in Tables 2, 3 and 4.

**Table 2:** Distribution of medial meniscal tear patterns in patients with and without Baker’s cyst. Percentages for tear patterns are calculated among patients with a medial meniscal tear in each group. The bold value indicates statistical significance at p < 0.05

Medial Meniscus Tear Patterns	Baker’s Cyst (*n* = 54)	Without Baker’s Cyst (*n* = 120)	*p*-value
Horizontal Tear	14 (25.9%)	13 (10.8%)	**< 0.001**
Radial Tear	6 (11.1%)	15 (12.5%)	
Vertical Tear	4 (7.4%)	21 (17.5%)	
Bucket Handle Tear	4 (7.4%)	16 (13.3%)	
Complex Tear	26 (48.1%)	55 (45.8%)	

**Table 3:** Distribution of lateral meniscal tear patterns in patients with and without Baker’s cyst. Percentages for tear patterns are calculated among patients with a lateral meniscal tear in each group

Lateral Meniscus Tear Patterns	Baker’s Cyst (*n* = 15)	Without Baker’s Cyst (*n* = 32)	*p*-value
Horizontal Tear	3 (20%)	9 (28.1%)	0.643
Radial Tear	1 (6.6%)	3 (9.3%)	
Vertical Tear	1 (6.6%)	2 (6.2%)	
Bucket Handle Tear	1 (6.6%)	3 (9.3%)	
Complex Tear	9 (60%)	15 (46.9%)	

**Table 4:** Tear localizations for both menisci based on cooper classification. The bold value indicates statistical significance at p < 0.05

Variable	Baker’s Cyst (*n* = 77)	Without Baker’s Cyst (*n* = 276)	*p*-value
Tear Localizations Based on Cooper Classification	A0: 3 (4.3%) A1: 2 (2.9%) A2: 8 (11.6%) A3: 23 (33.3%) B0: 0 (0%) B1: 0 (0%) B2: 1 (1.4%) B3: 16 (23.2%) C0: 0 (0%) C1: 0 (0%) C2: 1 (1.4%) C3: 0 (0%) D0: 0 (0%) D1: 0 (0%) D2: 0 (0%) D3: 0 (0%) E0: 0 (0%) E1: 0 (0%) E2: 1 (1.4%) E3: 5 (7.2%) F0: 0 (0%) F1: 1 (1.4%) F2: 2 (2.9%) F3: 6 (8.7%)	A0: 8 (5.3%)) A1: 5 (3.3%) A2: 20 (13.2%) A3: 42 (27.6%) B0: 0 (0%) B1: 2 (1.3%) B2: 17 (11.2%) B3: 24 (15.8%) C0: 0 (0%) C1: 0 (0%) C2: 0 (0%) C3: 2 (1.3%) D0: 0 (0%) D1: 0 (0%) D2: 3 (2%) D3: 1 (0.7%) E0: 0 (0%) E1: 0 (0%) E2: 4 (2.6%) E3: 2 (1.3%) F0: 0 (0%) F1: 0 (0%) F2: 6 (3.9%) F3: 16 (10.5%)	**< 0.001**

Age was significantly associated with Baker’s cyst formation in the univariate analysis (*p* = 0.002 for both models); however, this association did not remain significant after adjustment in either multivariate model (tear type model: *p* = 0.254; Cooper classification model: *p* = 0.180).

Chondral lesions remained independently associated with Baker’s cyst formation in both multivariate models (tear type model: OR: 5.081; 95% CI: 2.469–10.458; *p* < 0.001; Cooper classification model: OR: 5.703; 95% CI: 2.652–10.412; *p* < 0.001).

In the multivariate model evaluating tear morphology, horizontal, radial, and complex medial meniscal tears were significantly associated with Baker’s cyst formation (horizontal tear: OR: 7.321; 95% CI: 2.921–18.349; *p* < 0.001; radial tear: OR: 3.380; 95% CI: 1.136–10.056; *p* = 0.039; complex tear: OR: 4.000; 95% CI: 1.907–8.394; *p* < 0.001).

**Fig. 5:**
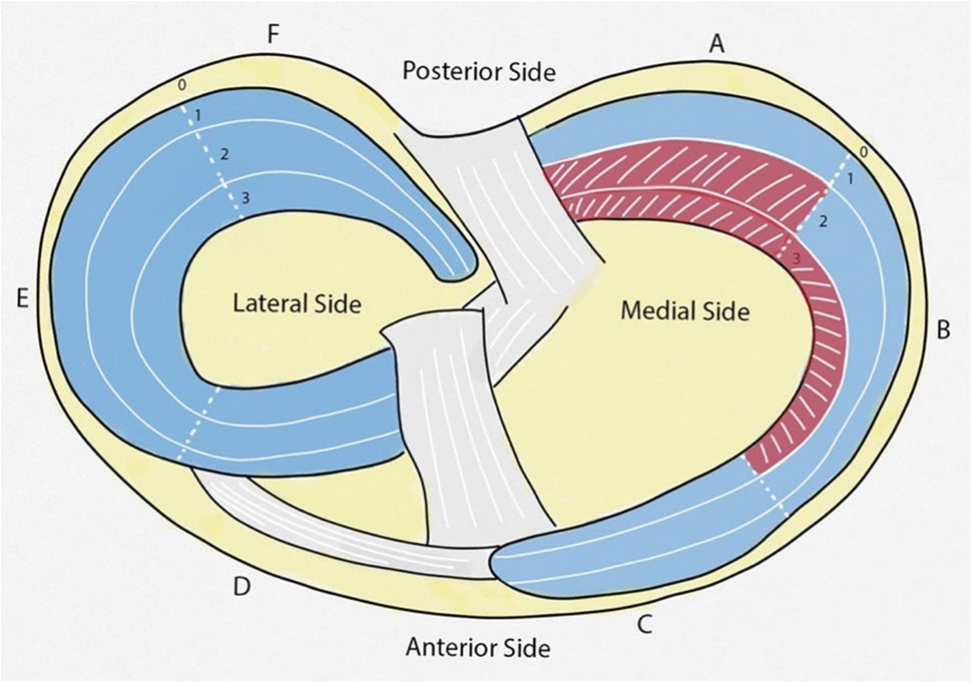
Tears involving the A2, A3, and B3 regions, as defined by the Cooper classification, are highlighted in the illustration and are significantly associated with Baker’s cyst development

In the multivariate model evaluating anatomical location, tears involving Cooper zones A2, A3, and B3 were independently associated with Baker’s cyst formation (A2: OR: 2.956; 95% CI: 1.133–7.713; *p* = 0.027; A3: OR: 3.978; 95% CI: 1.838–8.606; *p* < 0.001; B3: OR: 7.070; 95% CI: 2.878–17.371; *p* < 0.001). Tables 5 and 6 summarizes the univariate and multivariate logistic regression analyses for each tear type, tear location, and the additional variables described above. Among these, Cooper zones A3 and B3 demonstrated the strongest associations, indicating that posterior and central weight-bearing regions of the medial meniscus are most strongly associated with Baker’s cyst formation. An illustration of the areas involved is provided in Fig. 5.

**Table 5:** Univariate and multivariate logistic regression analyses evaluating the association between medial meniscal tear patterns and Baker’s cyst formation. Bold values indicate statistical significance at p < 0.05

		Univariate Logistic Model			Multivariate Logistic Model		
Variable	Reference Category	Odds Ratio	95% Cl	*p*-value	Odds Ratio	95% Cl	*p*-value
Age	-	1.031	1.011–1.050	**0.002**	0.985	0.960–1.011	0.254
Chondral Lesion	No chondral lesion	5.445	3.071-9,654	**< 0.001**	5.081	2.469–10.458	**< 0.001**
Medial Meniscal Tear Pattern	No medial meniscal tear	Horizontal tear: 8.007 Radial tear: 3.717 Complex tear: 4.715	Horizontal tear: 3.350- 19.138 Radial tear: 1.119–9.112 Complex tear: 2.445–9.090	Horizontal tear: **<0.001** Radial tear: **0.011** Complex tear: **<0.001**	Horizontal tear: 7.321 Radial tear: 3.380 Complex tear: 4.00	Horizontal tear: 2.921–18.349 Radial tear: 1.136–10.056 Complex tear: 1.907–8.394	Horizontal tear: **<0.001** Radial tear: **0.039** Complex tear: **<0.001**

**Table 6:** Univariate and multivariate logistic regression analyses evaluating the association between meniscal tear location based on the Cooper classification and Baker’s cyst formation. Bold values indicate statistical significance at p < 0.05

		Univariate Logistic Model			Multivariate Logistic Model		
Variable	Reference Category	Odds Ratio	95% Cl	*p*-value	Odds Ratio	95% Cl	*p*-value
Age	-	1.031	1.011–1.050	**0.002**	0.982	0.957–1.008	0.180
Chondral Lesion	No chondral lesion	5.445	3.071-9,654	**< 0.001**	5.703	2.652–10.412	**< 0.001**
Tear Location. Based On Cooper Classification	No meniscal tear	A2: 2.939 A3: 4.829 B3: 6.531	A2: 1.161–7.438 A3: 2.439–9.560 B3: 2.929–14.565	A2: **0.023** A3: **<0.001** B3: **<0.001**	A2: 2.956 A3: 3.978 B3: 7.070	A2: 1.133–7.713 A3:1.838–8.606 B3: 2.878–17.371	A2: **0.027** A3: **<0.001** B3: **<0.001**

To explore the secondary hypothesis, age, medial meniscal tear patterns, and tear locations were evaluated in relation to chondral lesions. Separate multivariate models were constructed for tear morphology and Cooper classification to account for collinearity among meniscal variables. Age remained significantly associated with chondral lesions in both models.

(both *p* < 0.001).

In univariate analyses, medial meniscal tear patterns and tear locations showed associations with chondral lesions similar to those observed for Baker’s cysts. Specifically, horizontal, radial, and complex tears, as well as tears located in the A2, A3, and B3 regions, were significantly associated with chondral lesions. However, these associations did not remain statistically significant in either multivariate model. The results of the statistical analysis evaluating chondral lesions in relation to meniscal tear types and locations are presented in Tables 7 and 8.

**Table 7:** Univariate and multivariate logistic regression analyses evaluating the association between medial meniscal tear patterns and chondral lesions. Bold values indicate statistical significance at p < 0.05

		Univariate Logistic Model			Multivariate Logistic Model		
Variable	Reference Category	Odds Ratio	95% Cl	*p*-value	Odds Ratio	95% Cl	*p*-value
Age	-	1.120	1.088–1.152	**< 0.001**	1.108	1.076–1.142	**< 0.001**
Medial Meniscal Tear Pattern	No medial meniscal tear	Horizontal tear: 3.231 Radial tear: 3.231 Complex tear:4.098	Horizontal tear: 1.313–7.950 Radial tear: 1.192–8.755 Complex tear: 2.156–7.786	Horizontal tear: **0.011** Radial tear: **0.021** Complex tear: **<0.001**	Horizontal tear: 2.038 Radial tear: 1.322 Complex tear: 1.767	Horizontal tear: 0.719-5.775 Radial tear: 0.412–4.238 Complex tear: 0.852–3.657	Horizontal tear: 0.181 Radial tear: 0.639 Complex tear: 0.126

**Table 8:** Univariate and multivariate logistic regression analyses evaluating the association between medial meniscal tear locations and chondral lesions. Bold values indicate statistical significance at p < 0.05

		Univariate Logistic Model			Multivariate Logistic Model		
Variable	Reference Category	Odds Ratio	95% Cl	*p*-value	Odds Ratio	95% Cl	*p*-value
Age	-	1.120	1.088–1.152	**< 0.001**	1.116	1.084–1.150	**< 0.001**
Tear Location. Based On Cooper Classification	No meniscal tear	A2: 2.498 A3: 3.568 B3: 2.660	A2: 1.042–5.990 A3: 1.777–7.165 B3: 1.142–6.197	A2:**0.040** A3:**<0.001** B3: **0.023**	A2:2.381 A3: 1.613 B3: 1.043	A2: 0.822–6.897 A3:0.726–3.586 B3: 0.404–2.690	A2: 0.110 A3:0.0.240 B3: 0.931

## Discussion

### Main findings

The main finding of this study is that specific meniscal tear patterns and locations were significantly associated with Baker’s cysts. Horizontal, radial, and complex tears were linked to cyst presence, whereas vertical and bucket-handle tears were not. Similarly, tears in the A2, A3, and B3 zones of the Cooper classification showed associations. These same tear patterns and locations were also related to chondral lesions in univariate analysis.

### Potential mechanisms linking meniscal pathology to Baker’s Cysts

There is limited research specifically examining how meniscal tears contribute to Baker’s cyst formation. Several mechanisms have been implicated in the pathogenesis of Baker’s cysts, with joint effusion being one of the primary factors [7, 14, 20, 25]. Specific tear types and their locations may contribute to Baker’s cyst formation by disrupting the normal function of the meniscus, potentially leading to effusion, long-term chondral damage and altered intra-articular fluid dynamics. Roemer et al. investigated the relationship between knee effusion and meniscal tears, reporting that knees with meniscal pathology exhibit significantly greater joint effusion compared to those without meniscal damage [28]. However, one study suggests that knee effusion is not causing Baker’s cyst formation, even though meniscal tears were found to significantly increase knee effusion in the same study [3]. Taken together, these findings may suggest that Baker’s cyst formation is likely driven by multiple pathophysiological mechanisms rather than a single factor.

Synovial inflammation secondary to meniscal tears may represent an additional pathway by increasing chronic joint effusion and promoting the passage of excess intra-articular fluid into the gastrocnemius-semimembranosus bursa [21, 27]. However, this mechanism has not been extensively studied. Wu et al. reported that oblique and bucket-handle tears were associated with the most severe synovial inflammation, followed by radial and longitudinal tears, whereas horizontal tears induced the least inflammation. This pattern partly conflicts with our findings and further supports the view that the relationship between meniscal pathology and Baker’s cysts is likely multifactorial rather than driven by a single mechanism [37].

### Tear morphology, tear location, and chondral lesions

Horizontal tears are a common degenerative tear type and are frequently associated with chondral changes and osteoarthritis at long-term follow-up, this may explain their significant association with Baker’s cyst formation in our study [1, 15]. As horizontal tears are more commonly seen in older populations, which may also contribute to Baker’s cyst formation [22]. Horizontal meniscus tears are associated with altered biomechanics and Beamer et al. reported that medial meniscus horizontal tears result in increased contact pressures across all flexion angles in a cadaveric model, emphasizing the biomechanical severity of this tear type and its potential to accelerate joint degeneration [4].

Radial tears compromise the meniscus’s ability to distribute loads effectively by disrupting the conversion of axial loads into hoop stresses. Bergkvist et al. noted that radial tears of the medial meniscus, particularly in the posterior horn, exhibit the highest association with ipsilateral cartilage damage compared to other morphologic tear types [5]. Wu et al. further reported that cartilage damage was more severe in radial tears than in horizontal tears [36]. These biomechanical factors may explain the association of radial tears with Baker’s cyst formation. However, it is important to distinguish between true radial tears and root tears, as many posterior radial tears are in fact root tears. Root tears have particularly detrimental biomechanical consequences, often leading to rapid cartilage degeneration and early progression to osteoarthritis, which may further contribute to Baker’s cyst development [13]. In the present study, root tears were not evaluated as a separate variable, and some posterior radial tears may have included undifferentiated root injuries.

Vertical tears, which run parallel to the meniscus fibers, generally do not compromise the meniscus’s ability to convert axial loads into hoop stresses. This could explain why vertical tears were not associated with Baker’s cysts in our study [23].

Bucket-handle tears lack substantial biomechanical and clinical data regarding their effects on the knee joint. This tear type was not associated with Baker’s cyst formation in our study, likely due to its symptomatic and acute nature, which often necessitates immediate surgical intervention and relatively younger age which may contribute to the lack of association with Baker’s cyst formation [12].

Ramp lesions were also not associated with Baker’s cyst formation in our study [11]. Boonrad et al., in a cadaveric study, found that isolated unstable ramp lesions did not significantly affect force transmission, contact area, or contact pressure in the medial tibiofemoral joint [6]. The lack of association in our findings may be due to the acute and symptomatic nature of ramp lesions, similar to bucket-handle tears, which necessitates early intervention and relatively younger age demographic which may contribute to the lack of association with Baker’s cyst formation [26].

Age was significantly associated with Baker’s cyst presence in the univariate analysis but this effect did not remain significant in the multivariate model. This may suggest that the association between horizontal and complex tears and Baker’s cysts is not explained by age alone. However, because these tear types are commonly seen in more degenerative knees and older patients, residual confounding cannot be fully excluded. Therefore, the observed associations may reflect both biomechanical factors and age-related degenerative changes rather than a purely independent mechanical effect.

Although no prior studies specifically link Cooper zones A2, A3, or B3 to Baker’s cysts, the posterior medial meniscus is well known to be associated, and zones A2 and A3 correspond to this region [33]. The association of A2 and A3 with Baker’s cysts may partly relate to their posterior medial location near the gastrocnemius–semimembranosus bursa. However, anatomical proximity alone may be insufficient, as ramp lesions in the same region were not significantly associated. Regarding Cooper zone B3, Henry et al. reported that radial and complex tears in the inner third of the medial meniscus were significantly associated with chondral lesions. This may suggest that B3 tears, similar to A3 tears, may contribute to Baker’s cyst formation indirectly through cartilage damage [18].

Chondral lesions and horizontal, complex and radial tears were associated in univariate analysis, and these tear types were studied in different studies. Christoforakis et al. found that horizontal and complex tears were significantly linked to chondral lesions and Henry et al. reported that radial and complex tears were linked to chondral damage [8]. Our findings align with those of Henry et al. except for horizontal tears and are consistent with Christoforakis et al. in confirming the involvement of horizontal tears. These results may suggest that specific meniscal tear patterns may contribute to chondral lesions, which could indirectly influence the development of Baker’s cysts. Although no study has specifically examined Cooper zones in relation to chondral damage, their parallels with Baker’s cyst associations may indicate a shared underlying mechanism. Additionally, Rupp et al. highlighted that articular cartilage lesions were the intra-articular pathologies most frequently associated with popliteal cysts, underscoring the importance of chondral lesions in this context [29]. Saylik et al. investigated the relationship between the severity of chondral lesions and effusion in patients with Baker’s cysts, finding a significant association that underscores the critical role of chondral lesions in the pathogenesis of Baker’s cysts [31]. This may explain the mechanism by which meniscal tear patterns and locations are linked to Baker’s cyst formation. More studies are needed to understand the mechanism.

### Limitations

This study has several limitations that should be considered. First, the retrospective design and inclusion of patients operated on by two surgeons may limit generalizability. In addition, as the study population consisted exclusively of patients undergoing arthroscopic surgery, a degree of selection bias toward more symptomatic intra-articular pathology is likely. Second, although major confounders such as age and chondral lesions were incorporated into the multivariate models, certain variables—including BMI, lower limb alignment and detailed prior treatment history—were not consistently available and could not be analyzed. Furthermore, meniscal tear morphology and anatomical location represent interrelated aspects of the same pathology, introducing potential multicollinearity. This was addressed by constructing separate multivariate models; however, residual interdependence between variables may still have influenced the estimates.Third, the cross-sectional nature of the study precluded assessment of lesion chronicity. The duration of meniscal tears and associated intra-articular pathology may play an important role in the development of chondral lesions and Baker’s cysts, and this temporal relationship could not be evaluated. Future longitudinal studies are required to clarify these time-dependent mechanisms. Fourth, joint effusion and synovial inflammation, which are considered important factors in the pathophysiology of Baker’s cyst formation, were not systematically assessed. These variables may represent intermediate mechanisms linking meniscal pathology to cyst development and should be evaluated in future MRI-based studies. Finally, some Cooper classification subgroups contained relatively small sample sizes, which may have reduced statistical power and limited the stability of subgroup analyses. In addition, the inclusion of dominant tear locations in cases with multiple lesions may have introduced classification bias, potentially affecting the precision of location-based analyses.

## Conclusion

This study found that horizontal, radial, and complex meniscal tear patterns, particularly those located in Cooper zones A2, A3, and B3, were significantly associated with Baker’s cyst presence. In univariate analysis, these tear types and locations were also associated with chondral lesions, suggesting possible shared or overlapping mechanisms. These findings may help identify pathological indicators associated with Baker’s cyst formation and may inform future research on the underlying mechanisms.

## Data Availability

The datasets used and/or analysed during the current study are available from the corresponding author on reasonable request.

## Abbreviations

ACL: Anterior cruciate ligament
CI: Confidence interval
MRI: Magnetic resonance imagining
OR: Odds ratio
BMI: Body mass index
